# Comparative Analysis of Serum C-reactive Protein and Procalcitonin as Diagnostic Markers in Odontogenic Infections: A Cross-Sectional Study

**DOI:** 10.7759/cureus.117034

**Published:** 2026-09-27

**Authors:** Arani Roy, Soumyabrata Sarkar, Subhadeep Maity, Mainak Datta, Madhurima Manna, Medha Mukherjee

**Affiliations:** 1 Department of Dentistry, Balurghat District Hospital, Balurghat, IND; 2 Department of Oral Medicine and Radiology, Haldia Institute of Dental Sciences and Research, Haldia, IND

**Keywords:** biomarkers, c-reactive protein, inflammation, odontogenic infections, procalcitonin

## Abstract

Introduction

Odontogenic infections produce varying degrees of local and systemic inflammatory response. Objective biomarkers may provide additional information regarding infection severity. This study compared the serum C-reactive protein (CRP) and procalcitonin (PCT) levels between patients with different odontogenic infections and healthy individuals.

Materials and methods

This cross-sectional comparative observational study included 90 participants: 60 patients with odontogenic infections and 30 healthy controls. The case group included patients with pulpitis, apical periodontitis, periapical abscess, periodontal abscess, or odontogenic space infection (12 participants each). Serum CRP and PCT concentrations were estimated using an enzyme-linked immunosorbent assay (ELISA). Data were analyzed using the independent-samples t-test, chi-square (χ²) test, one-way analysis of variance, and Tukey's honestly significant difference (HSD) post hoc test.

Results

Mean serum CRP was significantly higher in cases than controls (9.11 ± 9.50 versus 1.50 ± 0.50 mg/L; p < 0.001; Cohen's d = 0.98). Similarly, PCT was significantly higher in cases (1.43 ± 0.73 versus 0.15 ± 0.05 ng/mL; p < 0.001). CRP differed significantly among infection subgroups (p < 0.001), with the highest level in space infection (27.69 ± 1.17 mg/L). PCT also differed significantly (p < 0.001), reaching the highest level in space infection (2.70 ± 0.20 ng/mL).

Conclusion

CRP and PCT levels may serve as useful adjunctive biomarkers for assessing odontogenic infections and identifying extensive infections, particularly space infections.

## Introduction

Odontogenic infections are among the most common infectious conditions encountered in dental practice and may arise from pulpal, periapical, periodontal, or other dental sources. Although many odontogenic infections remain localized, progression into the surrounding fascial spaces can result in extensive tissue involvement and, in severe cases, systemic inflammatory response, sepsis, and potentially life-threatening complications [1]. Therefore, the early recognition of the severity and systemic involvement of odontogenic infections is important for appropriate clinical management. Clinical examination and conventional radiographic investigations remain fundamental for diagnosis; however, these methods may not always adequately reflect the degree of the systemic inflammatory response associated with infection [1,2].

C-reactive protein (CRP) is an acute-phase inflammatory protein that increases in response to tissue injury and infections. Owing to its rapid response to inflammatory stimuli, serum CRP has been widely investigated as a marker of infection and disease severity [3]. In odontogenic infections, elevated CRP concentrations have been associated with increased severity, particularly in infections extending beyond the localized dental tissues [4,5]. However, CRP is a nonspecific marker that may be elevated in several inflammatory and infectious conditions, thereby limiting its diagnostic specificity.

Procalcitonin (PCT), a precursor of calcitonin, is normally present in very low concentrations in healthy individuals. During significant bacterial infections and systemic inflammation, its production increases substantially in response to bacterial endotoxins and inflammatory mediators [6]. PCT has consequently gained attention as a potential biomarker for identifying bacterial infections and systemic inflammatory involvement [7]. Previous studies have demonstrated increased PCT levels in patients with odontogenic and maxillofacial infections, particularly in those with systemic inflammatory manifestations [4,8].

Despite the increasing interest in these biomarkers, their comparative value across different types of odontogenic infections remains insufficiently characterized. This study aimed to compare serum C-reactive protein (CRP) and procalcitonin (PCT) concentrations between patients with odontogenic infections and healthy individuals. The primary objective was to compare serum CRP and PCT concentrations between the infection and control groups. The secondary objective was to compare serum CRP and PCT concentrations among different types of odontogenic infections.

## Materials and methods

Study design and setting

This study was designed as a cross-sectional comparative observational study and was conducted in the Department of Oral Medicine and Radiology of Haldia Institute of Dental Sciences and Research, Haldia, India, over a period of one year and six months between January 2021 and June 2022. Participants were recruited from the outpatient departments of oral medicine and radiology.

Study population

A total of 90 participants were included in the study, comprising 60 patients with clinically and radiographically diagnosed odontogenic infections and 30 healthy controls. The case group consisted of five equal subgroups: pulpitis (n = 12), apical periodontitis (n = 12), periapical abscess (n = 12), periodontal abscess (n = 12), and odontogenic space infection (n = 12). The control group comprised 30 individuals with regular oral hygiene and no clinically evident dental or periodontal disease or orofacial pain. Participants were aged 16-80 years. Cases and controls were selected to ensure comparability with respect to age and sex. For analysis, participants were categorized into four age groups: 16-31, 32-47, 48-63, and 64-80 years.

Sample size estimation

The sample size was estimated a priori using G*Power software version 3.1 (Heinrich-Heine-Universität Düsseldorf, Düsseldorf, Germany). The calculation was based on an effect size of 0.56 for serum CRP levels reported in a previous study of odontogenic infections [9]. A two-tailed independent-samples t-test was used, with an alpha error of 5% (α = 0.05), a statistical power of 80% (1 - β = 0.80), and a case-to-control allocation ratio of 2:1. The calculation yielded a minimum required sample size of 60 cases and 30 healthy controls, for a total sample size of 90 participants.

Eligibility criteria

Patients aged 16-80 years with odontogenic infection were eligible for inclusion. The infection categories included pulpitis, apical periodontitis, periapical abscess, periodontal abscess, and odontogenic space infection. The diagnosis was required to be established both clinically and radiographically. Healthy controls were required to have regular oral hygiene and no apparent dental or periodontal disease or orofacial pain.

Pulpitis was diagnosed based on clinical findings consistent with pulpal inflammation and appropriate pulp sensibility responses without evidence of apical abscess. Apical periodontitis was identified by tenderness to percussion and/or apical radiographic changes in association with a diseased or necrotic pulp. Periapical abscess was defined by the localized purulent infection of periapical tissues associated with a non-vital tooth. Periodontal abscess was defined as a localized purulent infection involving the periodontal tissues, typically associated with a periodontal pocket and localized swelling. Odontogenic space infection was defined as infection extending beyond the immediate dental tissues into the adjacent fascial spaces, based on clinical and radiographic findings.

Patients with systemic diseases or those undergoing treatment for systemic diseases were excluded. Patients who had received antibiotic therapy for odontogenic infection within the preceding 72 hours were excluded to minimize the potential influence of antimicrobial treatment on inflammatory biomarker concentrations. Patients with clinically and radiographically diagnosed inflammatory odontogenic cysts or granulomas were excluded from the study. Female participants who were pregnant, lactating, or taking oral contraceptive pills were excluded. Patients who were completely edentulous and individuals who were unwilling to participate were excluded.

Clinical and radiographic assessment

All participants underwent a clinical examination to establish the presence or absence of odontogenic disease. Patients presenting with dental pain or suspected odontogenic infections were evaluated clinically and subsequently assessed radiographically to confirm their diagnosis. The study included pulpal and periapical conditions, as well as periodontal abscess and odontogenic space infection. In this study, intraoral periapical radiographs were documented for patients with pulpitis and periapical disease, whereas panoramic radiographs were documented for periodontal abscesses and other odontogenic conditions. The final diagnosis was based on a combination of clinical and radiographic assessment.

Blood sample collection and serum preparation

For biochemical analysis, 2 mL of venous blood was collected from the antecubital fossa of each participant via venipuncture, using a 23-gauge needle. Blood samples were collected in the morning after an overnight fast of at least 12 hours. The blood was collected in disposable evacuated tubes and allowed to clot for 2 hours at room temperature or overnight at 4°C. The samples were subsequently centrifuged at 1000 revolutions per minute (rpm) for 20 minutes at 2°C-8°C. The separated serum supernatant was collected to estimate CRP and PCT levels. The samples were assayed within seven days when stored at 4°C, and when longer storage was necessary, samples were divided and stored at -20°C for up to one month or at -80°C for up to three months.

Estimation of serum CRP

Serum CRP concentration was determined using a human CRP enzyme-linked immunosorbent assay (ELISA) kit (Elabscience Biotechnology, Inc., Houston, TX), based on a quantitative sandwich ELISA. The manufacturer's assay format was 96T/48T with an assay time of approximately 3.5 hours. Serum, plasma, and other biological fluids were used as sample types, and 100 μL of each sample was used for the assay. The assay had a detection range of 0.39-25 mg/L, with a sensitivity of 0.23 mg/L and intra-assay and inter-assay coefficients of variation below 10%. Samples with concentrations exceeding the upper limit of the analytical range were appropriately diluted, and the final concentrations were calculated by applying the corresponding dilution factor.

The CRP assay was based on the sandwich ELISA principle. Microplate wells were pre-coated with antibodies specific for human CRP. Standards or serum samples were added to the wells, followed by a biotinylated detection antibody and avidin-horseradish peroxidase (HRP) conjugate. After incubation and washing to remove the unbound components, a substrate reagent was added to produce a color reaction in the wells containing the target antigen. The reaction was terminated using a stop solution, and the optical density was measured spectrophotometrically at 450 ± 2 nm. CRP concentrations were determined by comparing the optical densities of the samples with the standard calibration curve.

For the assay, 100 μL of the standard or serum sample was added to the wells and incubated for 90 minutes at 37°C. After removing the liquid, 100 μL of biotinylated detection antibody working solution was added, and the mixture was incubated for 60 min at 37°C. The plate was washed thrice, followed by the addition of 100 μL of horseradish peroxidase (HRP) conjugate working solution and incubation for 30 minutes at 37°C. The plate was subsequently washed five times, after which the stop solution was added and the plate was read at 450 nm. In healthy individuals, serum CRP concentrations are generally low, with reported values typically ranging from approximately 0.3 to 1.7 mg/L. Concentrations exceeding 10 mg/L are generally associated with an acute systemic inflammatory response. These values were used as the reference context for interpreting the measured serum CRP levels in this study.

Estimation of serum PCT

Serum PCT levels were estimated using a human PCT ELISA kit (Elabscience Biotechnology, Inc., Houston, TX). A quantitative sandwich ELISA was performed using 100 μL of the serum sample. The reported detection range was 31.25-2000 pg/mL, with a sensitivity of 18.75 pg/mL. The assay was based on specific antibody binding to human PCT, followed by the addition of a biotinylated detection antibody and avidin-HRP conjugate. After washing, the substrate was added, and the resulting color reaction was terminated with a stop solution. The optical density was measured at 450 ± 2 nm, and the PCT concentration was calculated from the corresponding standard curve.

For PCT estimation, 100 μL of standard or serum sample was added to each well and incubated for 90 minutes at 37°C. Following the removal of the liquid, 100 μL of biotinylated detection antibody working solution was added and incubated for 60 minutes at 37°C. The plate was washed three times, followed by the addition of 100 μL of HRP conjugate solution and incubation for 30 minutes at 37°C. After five additional washes, 90 μL of the substrate reagent was added and incubated for 15 minutes at 37°C. Finally, 50 μL of stop solution was added, and the optical density was measured at 450 nm. PCT is normally undetectable or present at very low concentrations in healthy individuals, with concentrations typically around 0.01 ng/mL. PCT concentrations above 0.5 ng/mL may be associated with systemic bacterial infection in the appropriate clinical setting.

Data collection and outcome measures

The principal outcome variables were serum CRP and PCT concentrations. The demographic variables included age and sex. The case group was further categorized according to the type of odontogenic infection, allowing the comparison of biomarker concentrations among pulpitis, apical periodontitis, periapical abscess, periodontal abscess, and odontogenic space infection. Each of the five diagnostic categories consisted of 12 participants.

Statistical analysis

The collected data were tabulated and analyzed using IBM SPSS Statistics version 25.0 (IBM Corp., Armonk, NY). The distribution of continuous variables was assessed using the Shapiro-Wilk test. Continuous variables are expressed as mean ± standard deviation, whereas categorical variables are summarized as frequencies and percentages. Continuous variables were compared between the case and control groups using independent-samples t-tests, while categorical sex distributions were compared using the chi-square (χ²) test. Differences in serum CRP and PCT concentrations among the five odontogenic infection subgroups were assessed using one-way analysis of variance (ANOVA), followed by Tukey's honestly significant difference (HSD) post hoc test for pairwise comparisons. The assumptions of normality and homogeneity of variance were assessed before parametric analyses. Equal subgroup sizes (n = 12 per infection category) were considered in the application of one-way ANOVA. Effect sizes were reported as Cohen's d for independent-samples t-tests and eta-squared (η²) for ANOVA. A two-tailed p-value of <0.05 was considered statistically significant.

Ethical considerations

This study was initiated only after obtaining approval (approval number HIDSR/IEC/2020/012, dated November 10, 2020) from the Institutional Ethics Committee of Haldia Institute of Dental Sciences and Research. Written informed consent was obtained from all participants before enrollment, and the confidentiality of participant information was maintained throughout the study.

## Results

A total of 90 participants were included in the study, comprising 60 (66.7%) patients with odontogenic infections and 30 (33.3%) healthy controls. The 60 patients with odontogenic infections were equally distributed among five diagnostic categories: pulpitis, apical periodontitis, periapical abscess, periodontal abscess, and odontogenic space infection, with 12 participants in each category.

Baseline characteristics

The mean age was 40.5 ± 12.3 years among patients with odontogenic infections and 38.2 ± 11.5 years among healthy controls. There was no statistically significant difference in age between the two groups (p = 0.386). Among patients with odontogenic infections, 34 (56.6%) were men, and 26 (43.4%) were women, compared with 18 (60.0%) men and 12 (40.0%) women among healthy controls. The difference in sex distribution was not statistically significant (p = 0.760). The corresponding effect sizes were small (Cohen's d = 0.190 for age and phi = 0.032 for sex), indicating similar demographic characteristics between the two groups (Table 1).

**Table 1:** Baseline demographic characteristics of the study population. Data are presented as mean ± standard deviation (SD) for continuous variables and frequency and percentage for categorical variables. An independent-samples t-test was used for age, and the chi-square (χ²) test was used for sex distribution; p < 0.05 was considered statistically significant. Effect sizes were presented as Cohen's d for independent t-tests and phi (φ) for chi-square distribution.

Variables		Cases, n = 60 (66.7%)	Healthy, n = 30 (33.3%)	Test statistic	P-value	Effect size
Age, years	Mean ± SD	40.5 ± 12.3	38.2 ± 11.5	t = 0.87	0.386	Cohen's d = 0.190
Sex, n (%)	Male	34 (56.6%)	18 (60.0%)	χ² = 0.09	0.760	φ = 0.032
	Female	26 (43.4%)	12 (40.0%)			

Comparison of serum CRP and PCT between cases and controls

Serum CRP concentrations were significantly higher among patients with odontogenic infections than among healthy controls (9.11 ± 9.50 versus 1.50 ± 0.50 mg/L; p < 0.001), with a Cohen's d of 0.98, indicating a large between-group effect. Similarly, serum PCT concentrations were significantly higher among patients with odontogenic infections than among healthy controls (1.43 ± 0.73 versus 0.15 ± 0.05 ng/mL; p < 0.001), with a Cohen's d of 2.14, indicating a very large between-group effect (Table 2).

**Table 2:** Comparison of serum inflammatory biomarkers between cases and healthy controls. Data are presented as mean ± standard deviation (SD). An independent t-test was used for between-group comparisons. Effect sizes were presented as Cohen's d for independent t-tests. *P < 0.05 was considered statistically significant. CRP: C-reactive protein

Parameters		Cases, n = 60 (66.7%)	Healthy, n = 30 (33.3%)	Test statistic	P-value	Effect size
Serum CRP, mg/L	Mean ± SD	9.11 ± 9.50	1.50 ± 0.50	t = 6.19	<0.001*	0.98
Procalcitonin, ng/mL	Mean ± SD	1.43 ± 0.73	0.15 ± 0.05	t = 13.50	<0.001*	2.14

Serum CRP according to the type of odontogenic infection

There was a highly significant difference in serum CRP concentrations among the five odontogenic infection subgroups (p < 0.001; η² = 0.989). The mean CRP concentration was lowest in patients with pulpitis (1.88 ± 0.12 mg/L), followed by apical periodontitis (3.45 ± 0.52 mg/L), periapical abscess (6.35 ± 1.43 mg/L), and periodontal abscess (6.18 ± 1.28 mg/L). The highest mean CRP concentration was observed in patients with odontogenic space infection (27.69 ± 1.17 mg/L). The 95% confidence intervals (CIs) for the mean CRP concentration ranged from 1.80-1.96 mg/L for pulpitis to 26.95-28.43 mg/L for odontogenic space infection (Table 3).

**Table 3:** Comparison of serum C-reactive protein concentrations according to odontogenic infection type. Data are presented as mean ± standard deviation (SD), with 95% confidence intervals (CIs). One-way analysis of variance (ANOVA) was used for calculating the F value with comparison among odontogenic infection types. *P < 0.05 was considered statistically significant. η², eta-squared effect size; CRP, C-reactive protein

Odontogenic infection type	Cases (n = 60)	Serum CRP (mg/L), mean ± SD	95% CI	Test statistic	P-value	Effect size (η²)
Pulpitis	12 (20%)	1.88 ± 0.12	1.80-1.96	F = 1252.7	<0.001*	0.989
Apical periodontitis	12 (20%)	3.45 ± 0.52	3.12-3.78			
Periapical abscess	12 (20%)	6.35 ± 1.43	5.44-7.26			
Periodontal abscess	12 (20%)	6.18 ± 1.28	5.37-6.99			
Space infection	12 (20%)	27.69 ± 1.17	26.95-28.43			

Serum PCT according to the type of odontogenic infection

Serum PCT concentrations also differed significantly among the five odontogenic infection subgroups (p < 0.001; η² = 0.832). The mean PCT concentration was 0.79 ± 0.06 ng/mL in patients with pulpitis, 1.12 ± 0.55 ng/mL in those with apical periodontitis, 1.27 ± 0.29 ng/mL in those with periapical abscess, and 1.25 ± 0.22 ng/mL in those with periodontal abscess. The highest mean PCT concentration was observed in patients with odontogenic space infection (2.70 ± 0.20 ng/mL). The 95% CIs for the mean PCT concentration ranged from 0.75-0.83 ng/mL for pulpitis to 2.57-2.83 ng/mL for odontogenic space infection (Table 4).

**Table 4:** Comparison of serum procalcitonin concentrations according to odontogenic infection type. Data are presented as frequency and percentage and mean ± standard deviation (SD), with 95% confidence intervals (CIs). One-way analysis of variance (ANOVA) was used for calculating the F value with comparison among odontogenic infection types. *P < 0.05 was considered statistically significant. η²: eta-squared effect size

Odontogenic infection type	Cases (n = 60)	Serum procalcitonin (ng/mL), mean ± SD	95% CI	Test statistic	P-value	Effect size (η²)
Pulpitis	12 (20%)	0.79 ± 0.06	0.75-0.83	F = 68.22	<0.001*	0.832
Apical periodontitis	12 (20%)	1.12 ± 0.55	0.77-1.47			
Periapical abscess	12 (20%)	1.27 ± 0.29	1.09-1.45			
Periodontal abscess	12 (20%)	1.25 ± 0.22	1.11-1.39			
Space infection	12 (20%)	2.70 ± 0.20	2.57-2.83			

Post hoc comparison of inflammatory biomarkers

Tukey's HSD post hoc analysis demonstrated significant differences in CRP concentrations between pulpitis and apical periodontitis (mean difference, -1.57 mg/L; p = 0.004), pulpitis and periapical abscess (-4.47 mg/L; p < 0.001), pulpitis and periodontal abscess (-4.30 mg/L; p < 0.001), and pulpitis and odontogenic space infection (-25.81 mg/L; p < 0.001). CRP concentrations also differed significantly between apical periodontitis and periapical abscess (-2.90 mg/L; p < 0.001), apical periodontitis and periodontal abscess (-2.73 mg/L; p < 0.001), and apical periodontitis and odontogenic space infection (-24.24 mg/L; p < 0.001). No significant difference was observed between periapical abscess and periodontal abscess (p = 0.995).

For PCT, concentrations differed significantly between pulpitis and periapical abscess (mean difference, -0.48 ng/mL; p = 0.004), pulpitis and periodontal abscess (-0.46 ng/mL; p = 0.006), and pulpitis and odontogenic space infection (-1.91 ng/mL; p < 0.001). However, the difference between pulpitis and apical periodontitis was not statistically significant (p = 0.068). Similarly, PCT concentrations did not differ significantly between apical periodontitis and periapical abscess (p = 0.735), apical periodontitis and periodontal abscess (p = 0.835), or periapical abscess and periodontal abscess (p = 0.999). In contrast, patients with odontogenic space infection had significantly higher PCT concentrations than those in each of the other infection subgroups (all p < 0.001) (Table 5).

**Table 5:** Tukey's HSD post hoc comparison of serum C-reactive protein and procalcitonin between odontogenic infection subgroups. Tukey's honestly significant difference (HSD) post hoc test was used following a one-way analysis of variance. *P < 0.05 was considered statistically significant. CRP: C-reactive protein

Pairwise comparison	Serum CRP (mg/L)			Procalcitonin (ng/mL)		
	Mean difference	T value	P-value	Mean difference	T value	P-value
Pulpitis versus apical periodontitis	-1.57	-3.72	0.004*	-0.33	-2.61	0.068
Pulpitis versus periapical abscess	-4.47	-10.60	<0.001*	-0.48	-3.80	0.004*
Pulpitis versus periodontal abscess	-4.30	-10.19	<0.001*	-0.46	-3.64	0.006*
Pulpitis versus space infection	-25.81	-61.19	<0.001*	-1.91	-15.12	<0.001*
Apical periodontitis versus periapical abscess	-2.90	-6.88	<0.001*	-0.15	-1.19	0.735
Apical periodontitis versus periodontal abscess	-2.73	-6.47	<0.001*	-0.13	-1.03	0.835
Apical periodontitis versus space infection	-24.24	-57.47	<0.001*	-1.58	-12.51	<0.001*
Periapical abscess versus periodontal abscess	0.17	0.40	0.995	0.02	0.16	0.999
Periapical abscess versus space infection	-21.34	-50.59	<0.001*	-1.43	-11.32	<0.001*
Periodontal abscess versus space infection	-21.51	-51.00	<0.001*	-1.45	-11.48	<0.001*

## Discussion

The present study demonstrated that both CRP and PCT levels were significantly associated with odontogenic infections, with concentrations differing substantially between healthy individuals and infected patients. These findings support the concept that systemic inflammatory biomarkers can provide additional information regarding biological responses to odontogenic infections. Although odontogenic infections are frequently localized, progression from pulpal or periodontal inflammation to abscess formation and fascial-space involvement can produce an increasingly pronounced host inflammatory response. Therefore, the observed differences between diagnostic categories suggest that biomarker concentrations may reflect, at least in part, the extent and severity of the infection process.

CRP levels demonstrated a progressive pattern across different forms of odontogenic infection, with the greatest elevation observed in patients with space infection. This finding is biologically plausible because CRP is an acute-phase protein synthesized predominantly by hepatocytes in response to inflammatory cytokines, particularly interleukin-6 [3,10]. Localized pulpal inflammation may produce only a limited systemic response, whereas abscess formation and extension into the fascial spaces involve greater tissue destruction, bacterial dissemination, and the activation of systemic inflammatory pathways [1,11]. Consequently, the particularly pronounced CRP response in space infection may reflect the greater inflammatory burden associated with the infection, extending beyond the immediate dental tissues.

The present findings are consistent with those of previous studies that have evaluated CRP levels in odontogenic space infections. Kaur et al. reported that the CRP level was associated with the clinical course of odontogenic space infection and suggested that it could be useful for monitoring disease progression and treatment response [9]. Similarly, Bali et al. demonstrated the usefulness of CRP in monitoring odontogenic space infections and reported that its clinical behavior is related to infection severity [12]. These observations support the present finding that CRP levels increase with the extent of odontogenic infections. More recent evidence has also demonstrated that serial CRP assessments may correspond to improvement in clinical manifestations following treatment, supporting its potential role as a monitoring biomarker rather than merely a diagnostic parameter [13].

PCT demonstrated a broadly comparable pattern, with the greatest elevation occurring in patients with space infection. Unlike CRP, PCT is more closely associated with bacterial stimulation and systemic inflammatory activation [7]. During significant bacterial infections, cytokine- and endotoxin-mediated pathways induce the production of PCT in tissues outside the normal thyroid-related pathway [14]. This biological behavior may explain why PCT becomes particularly informative when odontogenic infection progresses from a localized process to extensive or systemic involvement. Therefore, these findings support the potential complementary role of PCT alongside CRP, particularly in patients in whom systemic bacterial infection is suspected.

Previous studies have similarly highlighted the usefulness of PCT for severe odontogenic and maxillofacial infections. Kim and Lee reported that PCT was associated with the severity of odontogenic maxillofacial infections and demonstrated a useful diagnostic performance for identifying sepsis [8]. These findings support the present observation that PCT levels are particularly elevated in patients with extensive infection. Other investigations have also suggested that PCT may provide information regarding bacterial infections and systemic involvement, which is complementary to conventional inflammatory markers [4,15,16].

An important finding of the present study was that CRP and PCT levels did not behave identically across all the diagnostic categories. CRP could distinguish some of the earlier or less extensive inflammatory conditions, whereas PCT could not consistently discriminate between closely related localized infections. This difference may be explained by the biological kinetics of the two biomarkers. CRP is highly responsive to inflammatory cytokine stimulation and can increase even when infection remains relatively localized [3]. In contrast, PCT is more strongly associated with a significant bacterial burden and systemic inflammatory activation [7,17]. Therefore, a relatively modest localized infection may produce an appreciable CRP response without generating an equivalent PCT response.

Neither biomarker clearly distinguished periapical abscess from periodontal abscess. This observation is clinically reasonable because both conditions represent localized suppurative infections and may produce comparable degrees of tissue inflammation and bacterial stimulation [18,19]. Therefore, the anatomical origin of the infection may be less important for systemic biomarker production than the extent of the tissue involvement and spread. Conversely, the consistent distinction between spatial infection and other odontogenic infection categories suggests that both biomarkers may be particularly useful when infection extends into deeper anatomical spaces.

In the present study, serum CRP and PCT concentrations were significantly higher in patients with odontogenic infections than in healthy controls. Furthermore, both biomarkers varied significantly across the predefined infection categories, with the highest concentrations observed among patients with odontogenic space infections. These findings demonstrate differences in biomarker concentrations according to infection category but should not be interpreted as evidence that CRP or PCT independently measures infection severity or systemic inflammatory involvement. Because the study did not incorporate an independent standardized clinical severity scale or systemic inflammatory reference standard, the observed biomarker gradient should be regarded as an association with the predefined clinical categories rather than a validated measure of severity. Similarly, the present case-control comparison does not establish diagnostic accuracy. Although the findings may support the further investigation of CRP and PCT as adjunctive biomarkers in odontogenic infections, their clinical diagnostic utility would require prospective validation using appropriate diagnostic-performance analyses, including receiver operating characteristic curves, area under the curve, sensitivity, specificity, and clinically justified cutoff values.

Clinical implications

From a clinical perspective, CRP and PCT levels should not be considered replacements for detailed clinical examination, radiographic assessment, or clinical judgment. Rather, they may serve as adjunctive tools when the severity of infection is uncertain. In particular, markedly elevated levels of inflammatory biomarkers in patients with extensive swelling, fever, malaise, dysphagia, trismus, or other systemic manifestations could support closer observation and more urgent management. PCT may be particularly useful when systemic bacterial involvement or progression toward severe infection is suspected, whereas CRP may provide broader information regarding inflammatory activity and may be useful for subsequent monitoring.

Therefore, the potential clinical value of combining both biomarkers is greater than relying on either marker alone. CRP may provide sensitivity to inflammatory changes occurring during localized disease, whereas PCT may provide additional information regarding significant bacterial infection and systemic involvement. Such complementary information could assist clinicians in identifying patients who require intensive evaluation, specialist referral, hospital-based management, or close follow-up. However, biomarker concentrations should never be used independently to determine the need for antibiotics because antimicrobial decisions should be based on diagnosis, clinical severity, anatomical spread, systemic involvement, and established principles of odontogenic infection management.

Limitations

This study has several limitations. First, although the overall sample size was determined for the primary case-control comparison, the number of participants within each odontogenic infection subgroup was relatively small (n = 12), which limits the precision of subgroup estimates and the generalizability of comparisons among individual infection categories. Second, the single-center design and clinically selected study population may have introduced selection bias and may limit the generalizability of the findings to other clinical settings and patient populations. Third, the cross-sectional assessment of CRP and PCT at a single time point precludes the evaluation of temporal changes and limits conclusions regarding prognosis, treatment response, or causal relationships. Fourth, the study did not include an independent, standardized measure or reference standard for infection severity or systemic inflammatory status. Therefore, the observed differences in CRP and PCT concentrations should not be interpreted as establishing the diagnostic accuracy of these biomarkers or as independently identifying infection severity.

Although patients with systemic diseases and recent antibiotic use were excluded, residual confounding from other factors that may influence inflammatory biomarker concentrations, including smoking, obesity, recent inflammatory or infectious illness, periodontal inflammatory burden, and concomitant medications, cannot be excluded. The exclusion of oral contraceptive users may also have introduced additional selection considerations and was based on the predefined eligibility criteria. Finally, the large effect size observed for CRP across infection subgroups (η² = 0.989) and the small within-group standard deviations warrant verification against the original laboratory data. The assay units, detection ranges, dilution procedures, and concentration calculations were also reviewed to ensure consistency between the laboratory methodology and reported results. Further multicenter studies with larger subgroup samples, standardized severity measures, the assessment of relevant confounders, and formal diagnostic accuracy analyses are warranted to determine the clinical utility of CRP and PCT in odontogenic infections.

## Conclusions

Serum CRP and PCT concentrations were higher in patients with odontogenic infections than in healthy controls and varied across infection categories, with the highest concentrations observed in odontogenic space infections. These findings suggest that both biomarkers may provide adjunctive information regarding the inflammatory burden associated with odontogenic infections when interpreted alongside clinical and radiographic findings. However, the present study does not establish their ability to assess infection severity or diagnostic accuracy because an independent severity reference standard and formal diagnostic accuracy analyses were not performed. Larger multicenter studies incorporating standardized severity measures and serial biomarker measurements are warranted to further evaluate the diagnostic and prognostic utility of CRP and PCT in odontogenic infections.

## References

[1] Neal TW, Schlieve T (2022). Complications of severe odontogenic infections: a review. Biology (Basel).

[2] Barani M, Aliu X, Ajeti N, Asllani L (2023). Assessment of correlation between clinical, radiographic, microbiological, and histopathological examinations in identification of pulpal diseases - a single-centre study. Saudi Dent J.

[3] Sproston NR, Ashworth JJ (2018). Role of C-reactive protein at sites of inflammation and infection. Front Immunol.

[4] Lalmuanpuia C, Kaushik K, Kathuria B (2026). Inflammatory biomarker profiles in odontogenic infections: evaluation of C-reactive protein (CRP), procalcitonin, WBC count, and neutrophil-to-lymphocyte ratio. Cureus.

[5] Rosca O, Bumbu BA, Ancusa O (2022). The role of C-reactive protein and neutrophil to lymphocyte ratio in predicting the severity of odontogenic infections in adult patients. Medicina (Kaunas).

[6] Mućka S, Jakubiak GK, Pawlas N (2025). Procalcitonin: Infection or maybe something more? Noninfectious causes of increased serum procalcitonin concentration: updated knowledge. Life (Basel).

[7] Samsudin I, Vasikaran SD (2017). Clinical utility and measurement of procalcitonin. Clin Biochem Rev.

[8] Kim JK, Lee JH (2021). Clinical utility of procalcitonin in severe odontogenic maxillofacial infection. Maxillofac Plast Reconstr Surg.

[9] Kaur A, Sandhu A, Kaur T, Bhullar RS, Dhawan A, Kaur J (2019). Correlation between clinical course and biochemical analysis in odontogenic space infections. J Maxillofac Oral Surg.

[10] Ngwa DN, Pathak A, Agrawal A (2022). IL-6 regulates induction of C-reactive protein gene expression by activating STAT3 isoforms. Mol Immunol.

[11] LeJeune HB, Amedee RG (1994). A review of odontogenic infections. J La State Med Soc.

[12] Bali R, Sharma P, Ghanghas P (2017). To compare the efficacy of C-reactive protein and total leucocyte count as markers for monitoring the course of odontogenic space infections. J Maxillofac Oral Surg.

[13] Muhunzi D, Mrema E, Banda F, Mashauri HL (2026). Personalized interpretation of CBC and the role of CRP in early detection and monitoring of blood infection or sepsis: a diagnostic perspective. Health Sci Rep.

[14] Maruna P, Nedelníková K, Gürlich R (2000). Physiology and genetics of procalcitonin. Physiol Res.

[15] Simon L, Saint-Louis P, Amre DK, Lacroix J, Gauvin F (2008). Procalcitonin and C-reactive protein as markers of bacterial infection in critically ill children at onset of systemic inflammatory response syndrome. Pediatr Crit Care Med.

[16] Velur A, Varghese LJ, Kuruvila VE, Rahman S, Ukken NJ, James B (2025). Procalcitonin level compared to total white blood cell count and C-reactive protein in maxillofacial space infections - a prospective study. Ann Maxillofac Surg.

[17] Pawar VS, Shinde RV, Patil S (2026). Procalcitonin and C-reactive protein in septicaemia: correlation with bacterial profile and antimicrobial resistance in ICU setting. Cureus.

[18] Herrera D, Roldán S, González I, Sanz M (2000). The periodontal abscess (I). Clinical and microbiological findings. J Clin Periodontol.

[19] Siqueira JF Jr, Rôças IN (2013). Microbiology and treatment of acute apical abscesses. Clin Microbiol Rev.

